# Expression and regulatory asymmetry of retained *Arabidopsis thaliana* transcription factor genes derived from whole genome duplication

**DOI:** 10.1186/s12862-019-1398-z

**Published:** 2019-03-13

**Authors:** Nicholas L. Panchy, Christina B. Azodi, Eamon F. Winship, Ronan C. O’Malley, Shin-Han Shiu

**Affiliations:** 1 Genetics Program, Michigan State University, East Lansing, MI 48824 USA; 2 Department of Plant Biology, Michigan State University, East Lansing, MI 48824 USA; 3 Department of Biochemistry and Molecular Biology, Michigan State University, East Lansing, MI 48824 USA; 4 Department of Computational Mathematics, Science, and Engineering, Michigan State University, East Lansing, MI 48824 USA; 5 DOE Joint Genome Institute, Walnut Creek, CA 94598 USA; 6 Present address: NIMBioS, University of Tennessee, Claxton Bldg. 1122 Volunteer Blvd., Suite 106, Knoxville, TN 37996-3410 USA; 7 Present address: MYcroarray, 5692 Plymouth Rd, Ann Arbor, MI 48105 USA; 8 Plant Biology Laboratories, Michigan State University, 612 Wilson Road, Room 166, East Lansing, MI 48824-1312 USA

**Keywords:** Expression divergence, *cis-*regulatory evolution, Duplicate retention

## Abstract

**Background:**

Transcription factors (TFs) play a key role in regulating plant development and response to environmental stimuli. While most genes revert to single copy after whole genome duplication (WGD) event, transcription factors are retained at a significantly higher rate. Little is known about how TF duplicates have diverged in their expression and regulation, the answer to which may contribute to a better understanding of the elevated retention rate among TFs.

**Results:**

Here we assessed what features may explain differences in the retention of TF duplicates and other genes using *Arabidopsis thaliana* as a model. We integrated 34 expression, sequence, and conservation features to build a linear model for predicting the extent of duplicate retention following WGD events among TFs and 19 groups of genes with other functions. We found that TFs was the least well predicted, demonstrating the features of TFs are substantially deviated from duplicate genes in other function groups. Consistent with this, the evolution of TF expression patterns and cis-regulatory cites favors the partitioning of ancestral states among the resulting duplicates: one “ancestral” TF duplicate retains most ancestral expression and cis-regulatory sites, while the “non-ancestral” duplicate is enriched for novel regulatory sites. By modeling the retention of ancestral expression and cis-regulatory states in duplicate pairs using a system of differential equations, we found that TF duplicate pairs in a partitioned state are preferentially maintained.

**Conclusions:**

These TF duplicates with asymmetrically partitioned ancestral states are likely maintained because one copy retains ancestral functions while the other, at least in some cases, acquires novel *cis-*regulatory sites that may be important for novel, adaptive traits.

**Electronic supplementary material:**

The online version of this article (10.1186/s12862-019-1398-z) contains supplementary material, which is available to authorized users.

## Introduction

Plant genomes are replete with paralogous genes derived from a variety of duplication events and mechanisms, particularly whole genome duplication (WGD) [14, 15, 20, 21, 52, 56, 61, 72, 73, 77, 78]. Two ancient WGD events took place prior to the divergence of angiosperms [27]. Subsequently, more than a dozen WGD events have occurred across a variety of angiosperm lineages [33, 41, 47, 55, 65, 75]**,** including three in the lineage leading to *Arabidopsis thaliana* [9]. As the last known WGD event in the *Saccharomyces cerevisiae* [30, 79] and human [12, 53] lineages occurred prior to the radiation of angiosperms, WGD occurs much more frequently in plants relative to other eukaryotic lineages.

WGD accounts for ~ 90% of the expansion of TF families across plants lineages [42] and TFs are consistently enriched among WGD duplicates across divergent plant species [10, 36, 63]. In addition, plant TF duplicates derived from WGD are retained at higher rates than most plant genes with other functions [62, 63]. These duplicate TFs contribute significantly to plant adaption [34], agricultural traits [80], and domestication [39]. The expansion of several TF families coincides with major events in the evolution of plants, such as the migration to land and expansion of flowering plants [11, 64, 76]. TF duplication is also central to the evolution of flowering time [60], floral structures [67] and fruit development [38, 43].

Because WGD results in duplication of all genes in a genome, the differences in the degrees of expansion of different gene families [7, 24, 37, 62] must result from differential rates of gene retention. Previously, a collection of features including sequence properties (e.g. gene length), biochemical activities (e.g. expression level), evolutionary characteristics (e.g. substitution rates), and annotated functions have been used to assess the properties of retained duplicates in general [26, 45]. It remains an open question how well these properties may explain the retention rates of genes duplicated via different WGD events and in specific groups of genes, such as TFs and genes with other functions. It is also unknown how these properties differ between TFs and other functional groups of genes.

In this study, we first modeled the percent retention of TFs as a group and 19 other function groups of genes using 34 gene features in three broad categories (expression, sequence, and conservation). Then, to assess how the ancestral and extant functions of duplicate pairs have diverged relative to their ancestral function, we determined how gene expression and *cis-*regulatory sites of TF duplicates have likely evolved post WGD by inferring the ancestral expression and *cis-*regulatory states of extant TF duplicates. Finally, we modeled the evolution of TF WGD duplicates as a system of differential equations which tracks the change in frequency of duplicate pairs retaining the ancestral state in both, one, or neither to assess whether the partitioning of TF duplicates pairs is maintained by a bias against losing the ancestral state in the second duplicate copy.

## Results & discussion

### Retention of duplicate genes in different function groups following WGD

**Fig. 1:**
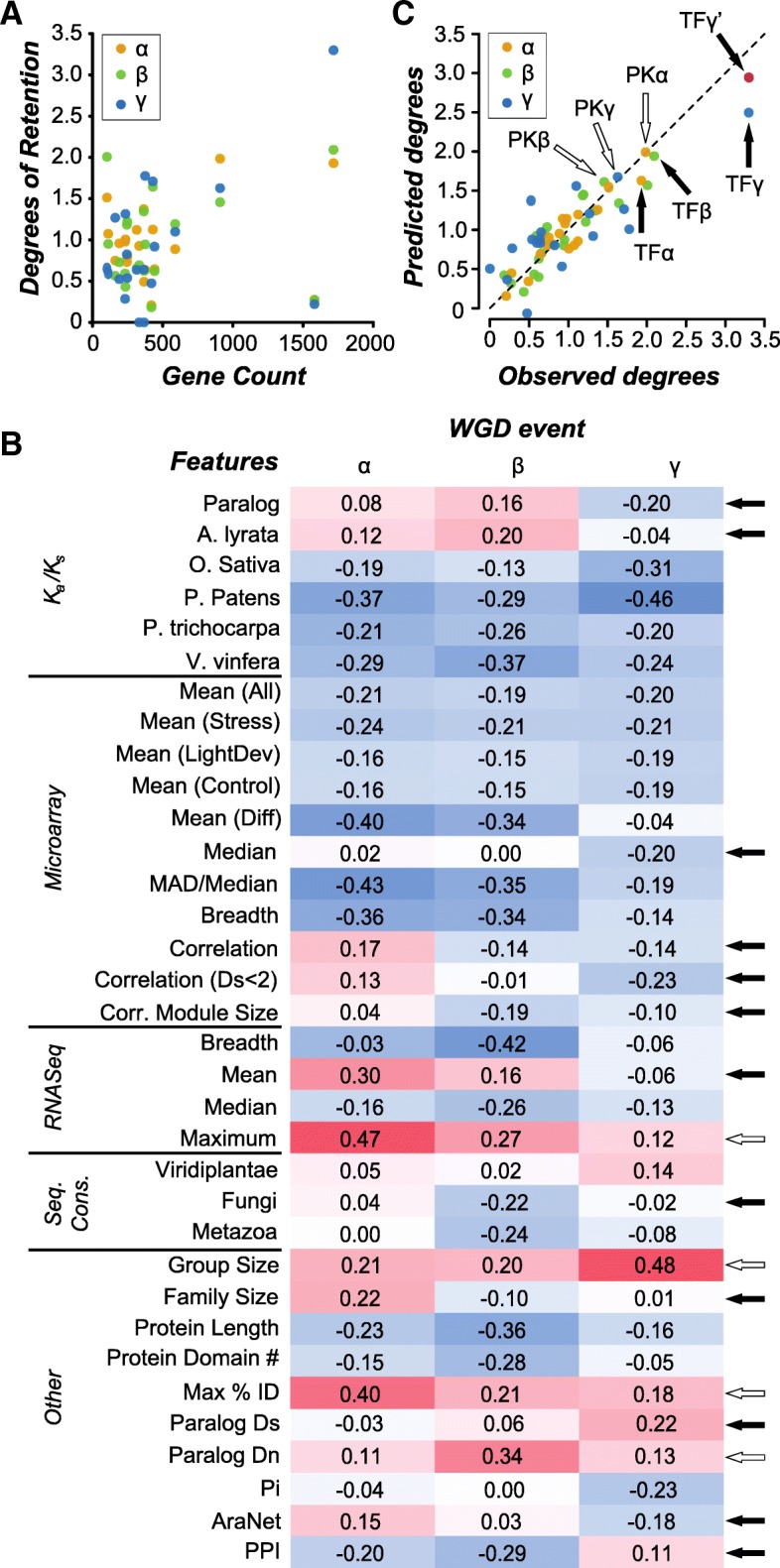
Linear model of the degree of duplicate retention in function groups based on genes features. **a** Relationships between gene counts and odds of retention of WGD duplicates across functional groups (α = orange, β = green, γ = blue). The correspondence between group sizes (numbers of genes) and degrees of retention (odds ratios) was determined using the square of the Pearson product-moment correlation coefficient (R^2^, α = 0.05, β = 0.16, γ = 0.04). **b** A heatmap of the Pearson product-moment correlation coefficient (PCC) between the values of a feature across different function groups (rows) and the odds of retention of functions groups from a particular WGD event (columns, indicated by the symbols α, β, and γ). Darker red: stronger positive correlation. Darker blue: stronger negative correlation. Features with different sign of correlation across WGD events are indicated by black arrows. Features with a large (≥0.20) difference in PCCs with the same sign are indicated by open arrows. **c** The observed odds of duplicate retention (x-axis) for each group plotted against the predicted odds of retention (y-axis) from the best model for each event (α = orange, β = green, γ = blue). Dotted line: equality between predicted and observed retention odds. Values from TFs are indicated by a black arrow while values from protein kinases are indicated by an open arrow. Red dot (TFγ’): the predicted odd ratio for TFs from the γ event after adjusting for difference in percent identity of TF genes. Performance of the models was assessed by calculating the R^2^ between the observed and predicted odds ratio for each event (α = 0.87, β = 0.83, γ = 0.65)

To assess the factors contributing to the differential retention of TF duplicates from WGD events and duplicates from WGD events involved in other functions, we first quantified the degree of duplicate retention of *A. thaliana* WGD duplicates in 20 different function groups. These function groups include TFs [28] and 19 other groups defined based on Gene Ontology (GO) molecular functions (see Methods, Additional file1: Table S1). The other functional groups were chosen based on their larger sizes for comparisons with TFs. Genes were classified as “WGD-duplicates” (both duplicate copies retained) or “WGD-singletons” (only one copy retained) depending on whether there were paralogs in corresponding duplicate blocks [9]. Because duplicate retention is expected to differ across different WGD events, duplicate pairs derived from the α, β, and γ WGD events [9] were analyzed separately. Here the duplicate retention (referred to as *R*_*d*_) is defined as ratio of the frequency of genes with WGD duplicates in each functional group to genome wide frequency of genes with WGD duplicates for each WGD event. Confirming results from earlier studies [42], among the 20 function groups examined, *R*_*d*_ values were highly heterogeneous and only TFs and protein kinases had significantly higher *R*_*d*_ than the genome average for all three WGD events (Additional file 2: Figure S1). Importantly, the difference in the *R*_*d*_ is not due to differences in gene number among functional groups alone (*R*^2^; α WGD = 0.05, β = 0.16, γ = 0.04; Fig. 1a).

With the *R*_*d*_ value defined, we next examined which gene features (sequence, expression, conservation, and others types, Additional file 1: Table S2) were correlated with *R*_*d*_ values among functional groups for each WGD event (Fig. 1b). We should emphasize that a subset of the features have been shown to be significantly associated with retention of WGD-duplicates as a whole [26]. Here we examined the relations for each WGD event independently. We found that, depending on the WGD event, the correlations between *R*_*d*_ and feature values can have different signs (black arrows, Fig. 1b) or magnitudes (white arrows, Fig. 1b), suggesting that, as WGD duplicates age, the mechanisms contributing their retention may differ (discussed in more details in the next section). To assess to what extent these features combined may predict *R*_*d*_, we fit a linear model that describes the relationship between the average feature values of genes in each function groups and *R*_*d*_ for each WGD event (Fig. 1c). Instead of using all 34 features, for each WGD event we focused on a subset of informative features (between 5 and 6 in each case) which maximized the F-statistic of the model (see Methods). Our models explained 87, 83, and 65% of the variance in degree of retention for the α, β and γ events respectively, significantly better than the null model (Table 1). Thus, the degrees of retention for duplicate genes in a function group can be predicted using the average expression, sequence, and conservation features.

**Table 1:** Statistics for the best fitting model for the odds ratio of duplicate retention for each WGD-event

WGD Event	# Features^a^	CoD^b^	F-statistic^c^	*p*-value^d^
α	6	0.87	13.8	5.6E-05
β	5	0.83	13.2	7.1E-05
γ	5	0.65	5.1	7.2E-03

^a^ The number of explanatory variables (features) used in the best fitting model

^b^ Coefficient of Determination (*R*^*2*^)

^c^ The F-statistic is a measure of the goodness of fit of the model to the observed odds ratio

^d^ The *p*-value of goodness of fit based on the F-statistic. A significant *p*-value (< 0.05) indicates that the model performs better than the null model by fitting the mean value to the data, after accounting for the number of features in the model

### Features explaining degrees of retention across function groups and WGD events

To assess the contribution of individual features in explaining the differences in *R*_*d*_ among function groups, we determined the change in explained variance caused by removing a feature from a model (Table 2). Important features that cause significant reduction in regression coefficients in the models tend to be those explaining degree of duplicate retention for all three WGD events. Examples include maximum expression level (RNA-seq), which positively correlated with retention, and mean expression level (microarray), which is negatively correlated with retention. This would suggest that functional groups with genes that have more specific expression patterns (i.e. lower average across all conditions, but higher maximum expression under a few specific conditions) increases the likelihood of duplicate retention. In addition to features important for all three WGD events, some features are more strongly correlated with retention of older duplicate genes. These features include lower nucleotide diversity and lower expression correlation, suggesting long term retention of duplicates favors genes experiencing stronger purifying selection and those with more divergent expression patterns (Table 2).

**Table 2:** The importance of all features used in the linear models of duplicate retention in function groups across each WGD event

Feature	Sign^a^	α^b^	β^b^	γ^b^
Expression Mean (AtGenExpress)	–	−0.29	− 0.09	− 0.49
Expression Maximum (RNASeq)	+	−0.56	− 0.59	− 0.14
Number of Domains	–	− 0.06	−0.36	n/a
Nucleotide Diversity (Pi)	–	−0.06	n/a	−0.32
Expression Correlation (AtGenExpress)	–	n/a	−0.24	−0.21
Expression MAD/Median (AtGenExpress)	–	−0.09	n/a	n/a
Protein Length (in Amino Acids)	+	−0.07	n/a	n/a
Paralog Dn	+	n/a	−0.07	n/a
Maximum Percent Identity	+	n/a	n/a	−0.2

^a^ The sign of the association between the feature and duplicate retention

^b^ Importance of features measured as the decrease in *R*^*2*^ when the feature is removed from the model, with more negative values indicating greater impact and therefore greater importance. An n/a indicates the feature was not used in the model for that event

However, certain parameters in our model do show sensitivity to what functional groups are used. In order to test the robustness of our models of duplicate retention, we made new, truncated data sets by leaving out one functional group per set and performed our optimization procedure again on each truncated set (see Additional file 1: Tables S3-S5). While most parameters show small deviation in response to the removal of individual functional groups (5–11% relative to the mean), we observed cases where the standard deviation was > 15%: the number of domains (15.7%) and nucleotide diversity (26.2%) in the α WGD model, as well as nucleotide diversity (15.1%) and maximum percent identity (16.5%) in γ WGD model. This elevated variance in these parameters when we used the truncated dataset is primarily driven by the removal of three functional groups: defense response, TFs, and translation. Of these, the TF group stands out as, without TFs, nucleotide diversity is dispensable in model of α WGD retention (F-statistic = 12.73 without nucleotide diversity, F-statistic = 12.74 with nucleotide diversity). In addition, our model fit of γ WGD retention is no longer significant after leaving TFs out (*p* = 0.12). This is expected given that estimates of TF retention are more underestimated in α and γ models than any other functional group (Fig. 1c) and thus the retention of TFs likely represents an extrema relative to most functional groups (Additional file 3: Figure S2).

Although the degree of retention predicted by the models closely align with the actual values for each function groups across each event (*R*^2^, α = 0.87, β = 0.83, γ = 0.65; see Fig. 1c), the estimates of different parameters is affected by the choice of functional groups being considered. The presence or absence of TFs in particular is highly influential which is to be expected given that TFs have such a high degree of duplicate retention. This is further demonstrated by the fact that the *R*_*d*_ of TFs was underestimated in all three models (black arrows, Fig. 1c, Additional file 3: Figure S2), and the net underestimation of TF retention summed across all models (*R*_*d*_ = 1.25) is 45.5% higher than the next nearest functional group (ubiquitin transferase, *R*_*d*_ = 0.86). For this reason, we chose to examine the feature distributions among TFs duplicates relative to those from other functional groups. Specifically, the retention of duplicates from the γ event is correlated with maximum percent identity, but the magnitude of the parameter associated with the feature is reduced by 37.9% if TFs are excluded (Additional file 1: Table S5). The identities of TF WGD-singletons (only one copy retained) to their best matches (66.9%) are significantly higher compared to the genome-wide WGD-singleton average (61.3%, Welch’s t-test, *p* = 1.9e-223), although identities of WGD-duplicates to the best matches are similar between TFs (71.3%) and genome-wide average (72.5%). The higher than average identity of TFs explains why the removal of TF functional group has such an impact on the γ model and the estimation of the effect of maximum percent identity in particular. In spite of this, the error in the γ model for TF retention was 0.802 even when TFs were included, the largest underestimation of all of our model predictions. However, if we assume TF WGD-singletons had a more typical distribution of maximum percent identity (i.e. duplicate are 10% higher on average than 5.6%) the predicted degree of TF retention of the γ event becomes 2.94 (red dot, Fig. 1c), reducing the error by almost half in our original model.

In addition to the linear models for predicting degrees of retention at the function group level, we have established machine learning models incorporating the same features to predict whether a gene likely have retained duplicate or not (Additional file 4: File S1). Similar to the linear model, the machine learning model performed the poorest when predicting TFs (Area Under Curve-Receiver Operating Characteristic = 0.75) compared to predicting all genes (0.88, Additional file 4: File S1). Taken together, we demonstrated that degree of retention for genes in different function groups are related to multiple features that are impacted by the timing of WGD events. However, while these features are useful for predicting the degree of retention for some function groups, they systematically underestimated degree of retention for TFs. The behavior of TFs departs from the norm in part because underlying differences in the features of TFs and genome average.

### Partitioning of ancestral expression states following TF duplication

**Fig. 2:**
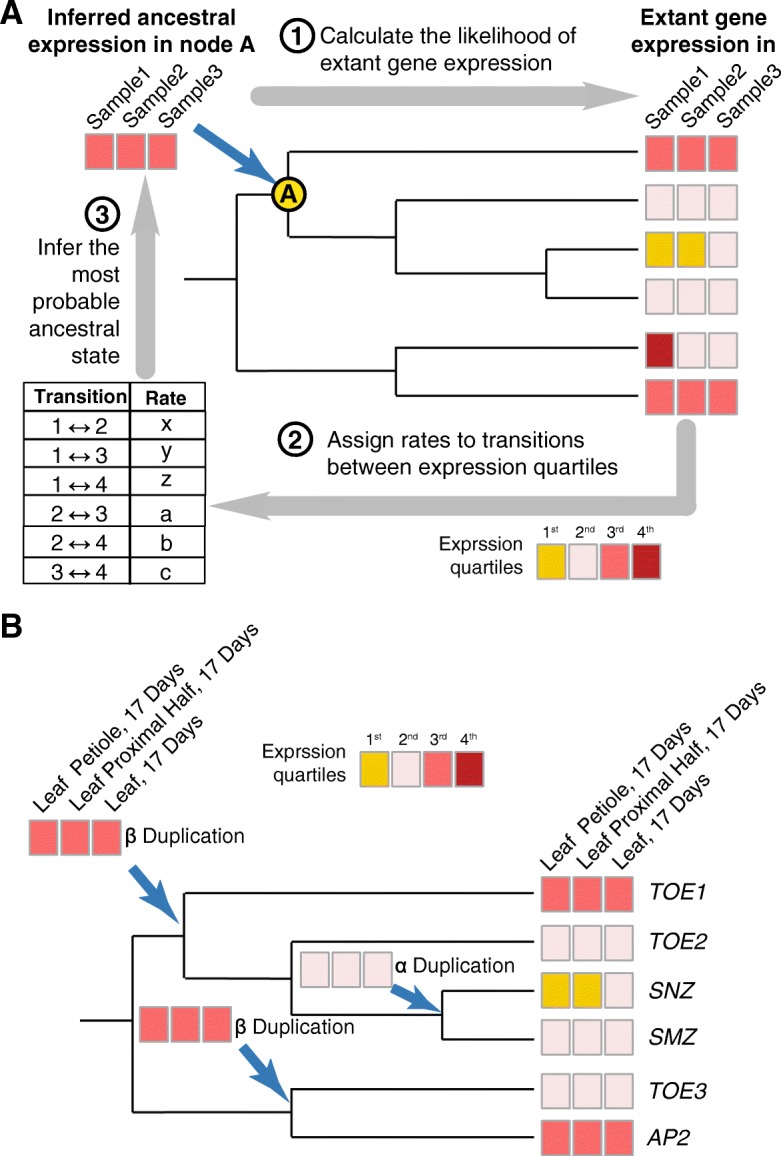
Inferring ancestral expression states. **a** An illustration of how BayesTraits is used to infer the most probable expression states at a given ancestral node on the evolutionary tree. First, evolutionary rates between expression states (defined by quartiles) are randomly assigned. These rates are then use to infer the most probable ancestral states from the observed extant expression values. Finally, the likelihood of the observed extant expression states is evaluated based on the current ancestral states and evolutionary rate and this likelihood is used to update estimations of the evolutionary rate. This process is repeated iteratively to optimize the evolutionary rates across the tree and thus the inferred ancestral states **b** The observed expression and inferred ancestral expression states of a branch of the AP2 domain family tree for three leaf developmental data sets. The ancestral states of the ancestors of TOE1 and TOE 2 (a β duplication), TOE3 and AP2 (a β duplication), as well as SNZ and SMZ (an α duplication) were inferred using the program described in (**a**)

To further explore what features retained TF WGD-duplicates possess, we examined how the expression patterns of retained TF WGD-duplicates have evolved following WGD events. Approaches to infer ancestral functions based on those of extant genes have been used to hypothesize the rate of gene activation and repression in duplicate genes in *Drosophila melanogaster* [49] and analyze the evolution of stress response in *A. thaliana* [40, 82]. Here we inferred the ancestral TFs expression prior to WGD using BayesTrait (see Methods), which assigns and optimizes rates of evolution based on sequence evolutionary rates in phylogenetic trees in order to determine the most likely ancestral state (Fig. 2). Expression data were grouped into four subsets and analyzed separately, including light and development sets (LightDev), control conditions (Ctrl), abiotic and biotic stress treatments (Stress), and differential expression between stress treatments and controls (Diff) (Additional file 1: Table S6). Ancestral expression values of 474 TF WGD-duplicate pairs were inferred from extant gene expression values discretized into quartiles (expression state = 0, 1, 2, or 3) using each expression data subset.

**Fig. 3:**
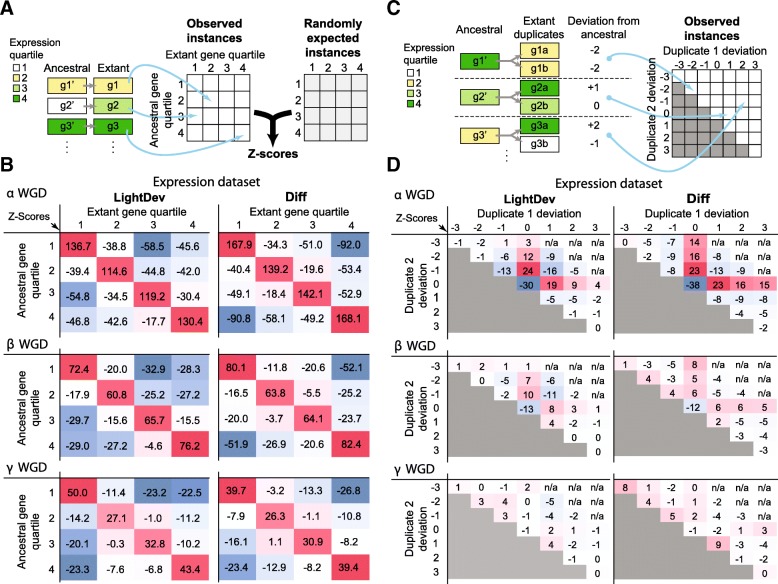
Evolution of expression in TF WGD-duplicates. **a** An illustration of how the z-scores in **b** are calculated. Individual TF duplicates were assigned to a cell using the extant (x-axis) and ancestral (y-axis) expression quartile values (dark green = 4th, green = 3rd, yellow = 2nd, white = 1st). Z-scores were then determined by comparing the frequency of the observed values to frequency distribution that would be expected if expression values were chosen randomly from a pool of extant and ancestral values. **b** Difference in expression quartile of individual TFs compared to their ancestors. Heatmaps show the z-scores of the observed frequency of each difference compared to the expected frequency for LightDev (left column) and Diff (right column) dataset in three WGD events (α = top, β = middle, γ = bottom). Darker red and blue indicates counts higher and lower than random expectation, respectively. **c** An illustration of how the z-scores in **d** were calculated. For each WGD TF duplicate, the difference in the expression quartile values (colored the same as in (**a**)) of an extant duplicate and its ancestral gene is defined as “deviation”. Duplicate 1 is the copy with a higher or equal expression quartile value compared to the other copy (duplicate 2). **d** Deviation values of pairs of TF WGD-duplicates. Heatmaps show the z-scores of the observed frequency of WGD-duplicate pair deviation compared to the expected frequency for LightDev (left column) and Diff (right column) datasets in three WGD events (α = top, β = middle, γ = bottom). Color correlates with the magnitude of the z-score as in (**a**)

To test how often the ancestral expression states of TFs were retained post-duplication, we compared the expression states of individual, extant TF WGD-duplicate to its inferred ancestral states (Fig. 3a). The most common ancestral-extant expression state combination for a TF was that the ancestral and extant TFs had the same expression quartiles (diagonal red boxes, Fig. 3b, Additional file 5: Figure S3), suggesting that most TF WGD-duplicates retain their original expression. However, when considering a pair of TF duplicates (Fig. 3c), the ancestral state was retained in only one duplicate more often than expected by chance (Fig. 3d). We should emphasize that the cases where both duplicates have the ancestral expression states are still more common (e.g. account for 53% of cases from the α-LightDev data set). However, under random permutation of duplicate pairs, 58% of α-duplicates in the LightDev data set are expected to be ancestral-ancestral (Additional file 1: Table S7). In contrast, we only expected 37% of pairs to be partitioned, but observed 45% pairs to have on ancestral and one non-ancestral expression states. We find the same trend using other data subsets (see Additional file 1: Table S7). Taken together, we found that, although TFs tend to preserve their ancestral expression states, the expression state evolution between a pair of TF duplicates tend to be “partitioned” with one ancestral and one non-ancestral copies.

The “partitioned” state of TF WGD-duplicates pairs is over-represented at lower degrees for more ancient β and γ WGD events (Fig. 3d). We confirmed that there is indeed significant interaction between the expression state of a TF WGD-duplicate pair and the timing of the WGD event (ANOVA, *p* < 2e-16), indicating that partitioning occurred relatively quickly after the most recent WGD, but that these partitioned patterns were not necessarily maintained as the duplicates age. Next we asked if TF duplicate expression levels tend to increase or decrease when they deviate away from the ancestral state using each expression data subset. For the LightDev (left panel, Fig. 3d), Ctrl, and Stress expression level subsets (Additional file 6: Figure S4), deviation from ancestral expression states among duplicates tend to be small (i.e. mostly by one quartile) and negative. In contrast, we found that TFs were equally likely to increase or decrease differential expression in response to stress compared to the ancestral state (Fig. 3d, Additional file 6: Figure S4). We also modeled the transition from ancestral expression (O) to higher (+) and lower (−) expression level states following WGD (see Methods). The results of these models can be found in (Additional file 7: Figure S5). In the two-parameter model (the rates from O to + and - were allowed to differ), the rate of evolution from O to - was 1.9~3.1 times more frequent than that from O to +. For the Diff subset, O to - was 1.2 times more frequent but not significant (*p* = 0.43). These results further suggest that the evolution of TF duplicates favors decreasing expression levels relative to the ancestral expression state. However, when looking at differential expression in response to stress, TF duplicates can evolve in either direction with similar likelihood. Thus, following duplication, TF duplicates may have increased or decreased responses to stress, rather than losing the response altogether, in sharp contrast to the patterns when all duplicate genes were considered [81, 82].

### Asymmetry in the partitioning of ancestral expression

**Fig. 4:**
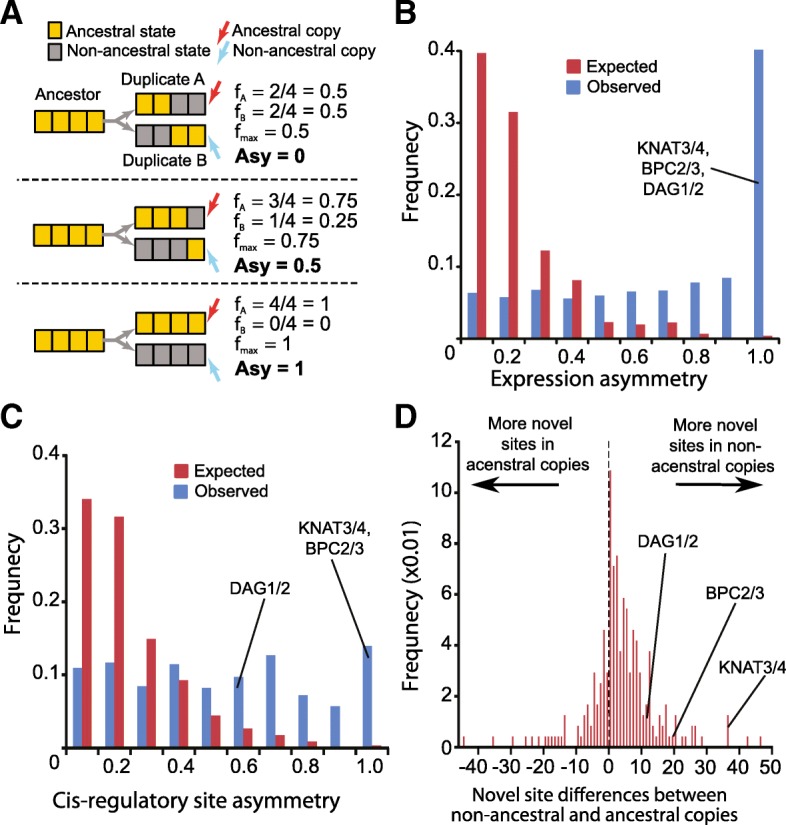
Asymmetry of ancestral state retention in TF WGD-duplicates. **a** Example of how Asymmetry score (Asy, see Methods) is calculated. Ancestral conditions are indicated by yellow boxes and non-ancestral conditions by grey boxes. Among a pair of duplicates, an ‘ancestral’ copy (red arrow) is the duplicate retains more ancestral states than the other, ‘non-ancestral’ copy (blue arrow). In case where equal numbers of ancestral states are inherited (the first case with Asy = 0), the ancestral and non-ancestral designation is assigned randomly. **b** The Asymmetry scores of ancestral expression partitioning between TF WGD-duplicates. Red columns indicate the expected frequency of each score bin based on a series of grouped Bernoulli trials (see Methods) while blue columns indicated the observed frequency. **c** The Asymmetry scores of ancestral *cis-*regulatory site partitioning between TF WGD-duplicates. Red and blue columns are as described in **(b)**. **d** The frequency distribution of the difference in number of novel *cis*-regulatory sites between ancestral and non-ancestral WGD duplicate copies. The value on the x-axis is calculated as the number of novel regulatory sites in the non-ancestral copy minus the number in the ancestral copy

Thus far we show that an ancestral expression state tends to be retained by only one copy of a TF WGD-duplicate pair when each expression state is considered individually. Considering that each gene will have multiple expression states (e.g. different tissue, developmental time point, environment), one outstanding question is whether each copy would retain different subsets or most of the ancestral expression states. To address this, we considered all expression data point with partitioned ancestral states between a pair of TF WGD-duplicates. We assume that the randomly expected number of ancestral states retained by a single WGD-duplicate follow a binomial distribution with a retention probability of 0.5 (both copy equally likely to retain a particular ancestral state). Next, we define the expected asymmetry of a duplicate pair as the difference in the fraction of ancestral states inherited between duplicates (mean = 0.18, Fig. 4a). The observed mean asymmetry between TF WGD-duplicates was 0.68, significantly higher than that from random partitioning (Welch’s t-test, *p* < 1e-323) (Fig. 4b). This biased partitioning was also found within each expression data subset (Additional file 1: Table S8). In addition, this biased partitioning of expression states between TF duplicates was not simply due to the use of correlated time course data because the mean asymmetry scores calculated using subsets of LightDev, Stress, and Diff conditions were virtually unchanged (Additional file 1: Table S8). Given these results, for each TF WGD-duplicate pair, we can generally define one duplicate as being “ancestral” and the other as being “non-ancestral”.

### Asymmetry in the partitioning of ancestral cis-regulatory sites

The ancestral copy is likely retained due to selection of inherited ancestral states. How about the non-ancestral copy? One possibility is that, despite the extreme asymmetry, some non-ancestral copies may still retain some ancestral functions that are subjected to selection. Another hypothesis is that the non-ancestral copy is retained because it has acquired a novel function in the form or new expression or regulatory states. To test this, we applied our model of ancestral-state partitioning to *cis-*regulatory sites. Using putative binding sites of 345 *A. thaliana* TFs [48], we inferred ancestral *cis-*regulatory sites of ancestral TFs (see Methods). Loss of an ancestral *cis*-regulatory site in only one TF copy (57%) occurs more often than expected (42.3%; t-test, *p* < 1e-323). In contrast, observed retention (10.5%, expected = 24.0%) and loss (16.2%, expected = 18.5%) of ancestral *cis-*regulatory sites in both WGD-duplicates were significantly less frequent than expected (*p* < 1e-323). In addition, the partitioning patterns of ancestral *cis-*regulatory sites were highly asymmetric (Kolmogorov–Smirnov test, *p* < 2.2e-16; Fig. 4c). Thus, much like what we observed for expression, TF WGD-duplicates can be classified into ancestral and non-ancestral copies with regard to *cis-*regulatory sites.

Most importantly, in 177 of the 249 duplicate pairs with ≥1 novel regulatory sites (71.0%), the non-ancestral copy tend to have more novel *cis*-regulatory sites (Fig. 4d), significantly higher than random expectation (50%, *p* < 3.8e-12). In addition, the novel *cis-*regulatory sites are only found in the non-ancestral copies in 61.8% of duplicate pairs, compared to 14% of pairs where all of the novel sites are in the ancestral copies. Novel *cis*-regulatory sites are also over-represented (odds ratio = 3.54) in the promoters of putative non-ancestral genes compared to ancestral ones (Fisher’s Exact Test, *p <* 2.2e-16). These patterns suggested that, the acquisition of novel *cis-*regulatory sites likely contribute to the retention of the non-ancestral TF duplicate copies. This conclusion is likely similar if we consider novel expression states because the ancestral and non-ancestral designation defined according to expression levels tend to have the same designation based on *cis*-regulatory sites (59.8%, compared to expected by random association at 24.6%, *p* = 1.8e-20).

**Fig. 5:**
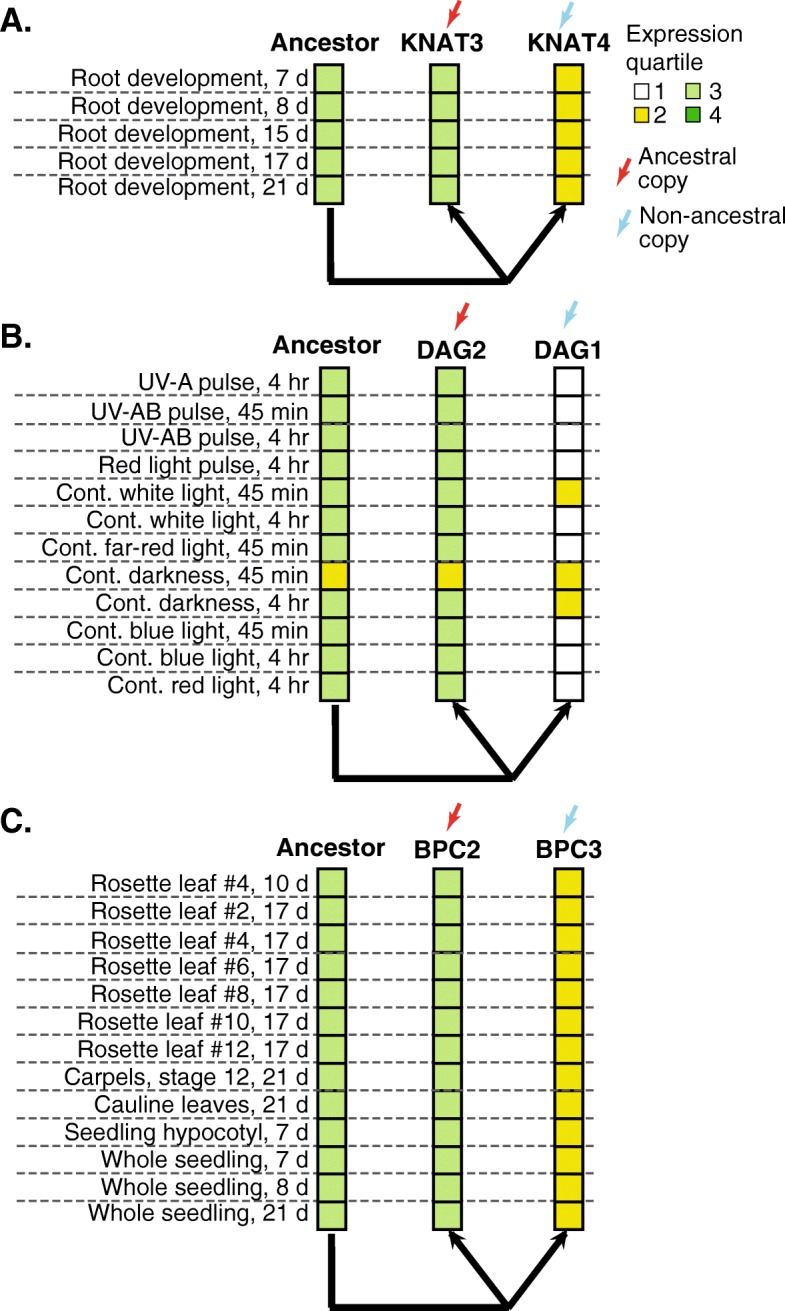
Expression partitioning between duplicate pairs with high regulatory asymmetry. Expression partitioning of three duplicate pairs KNAT3/4 (**a**), DAG1/2 (**b**), BCP2/3 (**c**) where the non-ancestral duplicate (blue arrow) exhibits differential function from the ancestral duplicate (red arrow). Expression quartile is indicated by color (dark green = 4th, green = 3rd, yellow = 2nd, white = 1st). Note that only expression conditions under which function differs between the duplicates are shown

For TF WGD-duplicates where the definition of ancestral and non-ancestral copies is supported by both expression and *cis-*regulatory data, we can find experimental evidence supporting functional divergence of duplicates. For example, KNAT3 and KNAT4 (Fig. 5a) function in different regions of the root [70, 71] while DAG1 and DAG2 (Fig. 5b) have opposite regulatory roles in control germination [23, 57]. While there is functional differentiation in the above cases, it is not clear what the ancestral function of the duplicates pairs was. However, for the pair BPC3/BPC2 (Fig. 5c), BPC3 functions antagonistically not only to BPC2, but other BPC family members as well, in controlling growth, leaf shape, and flower development [46]. Given that BPC2/BPC3 were duplicated during the β event, and only BPC1 diverged from BPC2 after WGD, it is therefore likely that BPC3 possess a novel function compared to the rest of the family.

### Patterns of WGD-duplicate divergences and partitioning results from evolutionary bias

**Fig. 6:**
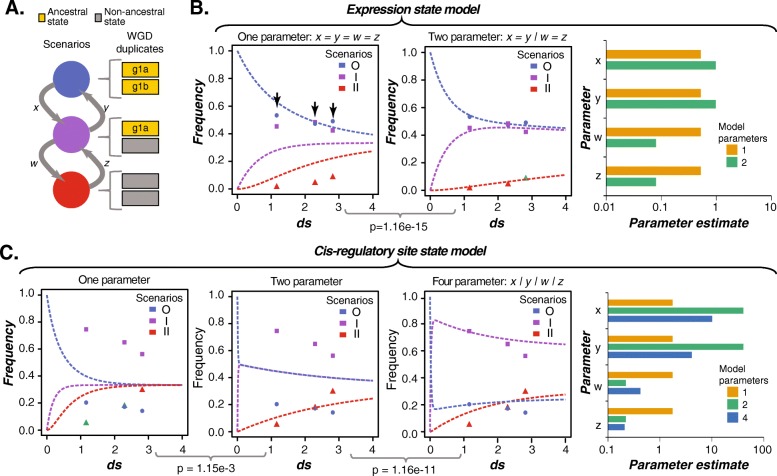
ODE models of TF WGD-duplicate expression and cis-regulatory site evolution relative to the ancestral state. **a** In this model, we consider the transition of WGD-duplicate pair expression states between three possible scenarios (O = both retained, I = one retained, II = neither retained) using four variables representing the rate of transition between state (*x,y,w,z*). **b** Left and middle: results for the one parameter (*x = y = w = z*) and two parameter (*x = y|w = z*) versions of the expression state model showing the change in time (x-axis) and the frequency (y-axis) of each scenario. Curves represent the continuous output of the model in different scenarios. The significance of including additional parameters (the *p*-value between the curly brackets) was determined using the likelihood ratio test. Right: A bar graph of the parameter values for the one (orange) and two (green) parameter versions of the expression ODE model. **c** Left three sub-graphs: results for the one-parameter (*x = y = w = z*), two-parameter (*x = y|w = z*), and four-parameter *(x|y|w|z*) versions of the *cis*-regulatory site model showing the change in time (x-axis) and the frequency (y-axis) of each WGD-duplicate-pair scenario. Curves represent the continuous output of the model in different scenarios. The *p*-values are derived from the likelihood ratio tests between models. Far right: a bar graph of the parameter values (*x,y,w,z*) for the one (orange), two (green), and four (blue) parameter versions for the *cis-*regulatory site ODE model

Partitioning of ancestral expression and regulation into ancestral and non-ancestral duplicates is favored following duplication of TFs. To determine if this ancestral state partitioning is maintained or if the partitioning is simply a transition state and eventually both copies would be lost, we modeled loss of ancestral states of TF WGD-duplicate pairs (see Methods). Using the synonymous substitution rate (*d*_*s*_) of TF WGD-duplicate pairs derived from the α, β, or γ events as a proxy for time, the rate of transition between WGD-duplicate pairs where neither (state O), only one (state I), or both (state II) duplicates had lost ancestral expression was modeled (Fig. 6a). We compared a model where the rates for losing the ancestral states in both duplicates were the same (one-parameter model) with a model where the O➔I transition rate was allowed to vary from that between I➔II (two-parameter model). These models were applied to all expression subsets with similar results and conclusions (Additional file 8: Figure S6). Below we discuss the LightDev subset as an example.

We found the two-parameter model to be significantly better at explaining the observed difference in WGD-duplicate states over time (Likelihood Ratio Test, *p* < 2e-14). Considering expression states, the O➔I transition rate were 7 to 13 times higher than the I➔II transition rate (Fig. 6b). Thus, the number of partitioned WGD-duplicates accumulated rapidly post WGD, followed by a relatively slow accumulation of cases where ancestral expression states had been lost in both duplicates. We also assessed a four-parameter model (O➔I, I➔II, II➔I, I➔O) of expression state evolution that was not better than the two-parameter model. In contrast, applying this same approach to model regulatory site evolution revealed that the four-parameter model is significantly better (*p* of 4.8e-13 and 1.2e-11 vs. one and two-parameter models, respectively; Fig. 6c). The rates governing the O➔I transition (*x)* are two orders of magnitude higher than the I➔II transition (*w,* Fig. 6d). Importantly, in the four-parameter model for *cis-*regulatory sites, there was a high rate of O➔I transition estimated at the early stage of WGD (blue curve, Fig. 6c). In addition, an appreciable proportion of partitioned duplicates lost ancestral regulatory sites in the second copy (green curve, Fig. 6c). This is in sharp contrast compared to the transition rate estimate over time for expression where second copies tend not to lose ancestral expression state (Fig. 6b), indicating that regulatory sites are faster evolving and more labile compared to expression states.

## Conclusions

In this study, we used linear models to assess how expression, conservation, and sequence structural features of genes in these functional groups may explain their retention rate difference. The value distributions of TF features are significantly different from genes in the rest of the genome that result in lower predictability. When considering each TF WGD-duplicate pair, one TF duplicate tends to have reduced expression level relative to the inferred ancestral level. In addition, we found that ancestral expression and cis-regulatory sites tends to be partitioned between TF duplicates asymmetrically such that there are distinct ancestral and non-ancestral duplicates. Interestingly, the non-ancestral TF duplicates tend to gain novel cis-regulatory sites that likely contribute to new expression patterns. Finally, we demonstrate a preference for maintaining partitioned expression and cis-regulatory site states between TF WGD-duplicate pairs.

Multiple mechanisms have been proposed to explain why duplicate genes are retained. The gene balance hypothesis [4–6] has been proposed to specifically explain the retention duplicates of TFs and other genes with larger numbers of interactions/functions [1, 42, 62, 63]. The hypothesis stipulates that duplicate genes with products that form multimeric complexes will tend to be retained to maintain the stoichiometry [5, 6] and enables future sub- and/or neofunctionalization [74]. We found that 7.5 and 13.9% of duplicates TF pairs have retained > 80% of ancestral expression in both copies in the Stress and LightDev data set respectively that may still be retained due to dosage balance. Nonetheless, most duplicates have substantially diverged expression patterns. For example, in > 60% cases (a case refers to a TF-WGD duplicate pair expressed in one of expression data subsets), ≥1 ancestral expression states are found uniquely in each duplicate. This partitioning of ancestral subfunctions between both duplicate copies is a hallmark of subfunctionalization [19], in which both duplicate copies are selected to maintain the full set of ancestral functions.

However, the partition of ancestral expression states is highly asymmetric in most cases. Although they can still be maintained by sub-functionalization, this asymmetry suggests that, if we assume that expression patterns can be treated as proxies of gene function, some TF WGD-duplicates take on only a small part of their ancestral functions and thus defined as non-ancestral. We found that the non-ancestral copies tend to have more novel *cis-*regulatory sites (Fig. 4d), suggesting that the gain of these novel sites may lead to neofunctionalization [50] or to escape from adaptive conflict [13], both of which involve the evolution of new or improved function that is selected for. The above observations are consistent with the suggestions that subfunctionalization may be a transition state to neofunctionalization [54]. The asymmetry may also suggest that the non-ancestral TF duplicate copies may be decaying functionally and are on their way to become pseudogenes, as suggested in a case study [35]. This can be due to genome fractionation/dominance, where one genome loses duplicates at a significantly higher frequency following WGD [59, 68].

To further improve our understanding of what roles all of these mechanisms play in TF duplicate retention will benefit from more detailed modeling of TF evolution. In this study, linear models for retention prediction and ODE models of ancestral expression and regulatory site evolution are based on WGD events that is > 50 million old. It will be crucial to consider data from other species with more recent WGD events to elucidate the early dynamics of TF evolution. In addition, we demonstrate that non-ancestral duplicates inherited fewer ancestral cis-regulatory sites tend to gain novel sites. It remains to be determined experimentally whether these novel sites control new expression patterns and, most importantly, is selected for rather than neutrally evolving. Finally, our study focuses on the overall pattern of TF evolution. It is anticipated that different TF families will evolve differently from each other. In future studies, it will be important to assess factors influencing retention for individual TF families.

## Methods

### Genome sequences, gene annotation, and expression data

Genome sequences, protein sequences, and gene annotation information for *A. thaliana* was obtained from Phytozome v10 (https://phytozome.jgi.doe.gov/pz/portal.html). WGDs were defined according to Bowers et al. [9] who used BLAST [3] to identify candidate duplicate gens in *A. thaliana*, with a hard Expect value cutoff of 1e-10. Duplicate pairs were used to identify syntenic regions and these regions were dated using by comparing duplicate pairs to orthologs from other species for which the time of divergence from *A. thaliana* had been estimated. Dating only employed pairs where matches between duplicates and orthologs were in > 35 aminio acids. Additionally, tandem genes in *A. thaliana* were defined as pairs of reciprocal best BLAST hits with an e-value <1e-10 and a threshold based on the number of annotated, non-homologous genes between the putative tandem duplicates (≤ 5 intervening genes, [24]). Expression microarray data for this study was taken from AtGenExpress [22, 31, 58], normalized using RMA [25] in R as performed previously [81]. The array data was divided into four groups: control conditions (in environmental condition experiments, Ctrl), light and development set (LightDev), abiotic and biotic stress treatments (Stress), and differential expression between stress treatments and controls (Diff) (Additional file 1: Table S9). The Diff data contains the log2 normalized difference between data sets for each stress condition/treatment/duration and its corresponding controls. In addition to microarray data, we have included a set of 214 RNA-sequencing samples (Additional file 1: Table S10) from *A. thaliana* Col1 wildtype from the Sequence Read Archive (https:// www.ncbi.nlm.nih.gov/sra) as of September 30, 2014. Raw sequence reads were processed using Trimmomatic [8], with a quality threshold of 20, window size of 4, and hard-clipping length of 3 for leading and trailing bases. Processed reads were then mapped to the *A. thaliana* genome using Tophat2 [32] and expression levels calculated with Cufflinks [69], both with a maximum intron length of 5000 bp.

### Defining TFs and other groups of genes in *A. thaliana*

TFs were defined according to the criteria used by the Plant Transcription Factor Database [28] with 1717 annotated TF loci in *A. thaliana*. To assess the degrees of TF duplicate retention after each WGD event, we defined a set of “functional groups” for comparison following from the procedure used in Maere et al. [42]. To compare among genes with divergent functions and to ensure the log odds indicative of the degrees of retention could be defined for each group, function groups were defined using Gene Ontology (GO) [2] terms in the molecular function and biological process categories from The *Arabidopsis* Information Resource (https://www.arabidopsis.org/), and only groups containing 100–2000 genes and ≥ 20 WGD-duplicate pairs were kept. We excluded GO:0006355 (regulation of transcription, DNA-templated) due to its substantial overlap with the TF group we have defined above. The remaining 19 function groups include: ATP Binding (GO:0005524), catalytic activity (GO:0003824), defense response (GO:0006952), DNA endoreduplication (GO:0042023), hydrolase activity hydrolyzing O-glycosyl compounds (GO:0004553), kinase activity (GO:0016301), lipid binding (GO:0008289), oxidoreductase activity (GO:001649), oxygen binding (GO:0019825), protein binding (GO:0005515), proteolysis (GO:0006508), response to auxin (GO:0009733), response to chitin (GO:0010200), RNA binding (GO:0003723), transferase activity, transferring glycosyl groups (GO:0016757), translation (GO:0006412), transporter activity (GO:0005215), ubiquitin-protein transferase activity (GO:0004842), zinc ion binding (GO:0008270). A list of genes in each group can be found in Additional file 1: Table S1.

### Fitting odds ratio of duplicate retention within each group of genes for each WGD event using linear models

A gene was designated as a “WGD-duplicate” if its paralog derived from a particular WGD event is present. For a gene without its paralog from WGD, it was designated as a “WGD-singleton” gene. The degree of retention for a function group, *g*, after a specific WGD event, *w*, is defined as:

$$ {R}_{g,w}=\frac{\left({D}_{g,w}/{S}_{g,w}\right)}{\left({D}_{\neg g,w}/{S}_{\neg g,w}\right)} $$

Where *D*_*g,w*_ and *D*_¬*g,w*_ are the numbers of WGD-duplicate genes in group *g* and those not in group g (¬g), respectively. *S*_*g,w*_ and *S*_¬*g,w*_ are the numbers of WGD-singleton genes in group *g* and those not in group g (¬g), respectively. The 95% confidence interval around the point-estimate *R*_*g,w*_ was defined using the “fisher.exact” function in R, the details of which can be found at in Fay [16]. For each WGD event, we established a general linear model with the glm function in the R environment which relates the *R*_*g,w*_ to a set of features of each gene group. The 34 features (predictor variables, Additional file 1: Table S2) were filtered with the following procedures to prevent over-fitting because we have only 20 function groups. We calculated the correlation between all features to find all cases where the absolute value of correlation was > 0.7. The considerations for which features to keep included: (1) how well each feature correlated with *R*_*g,w*_ on its own, (2) whether the feature was derived from a subset of another feature, and (3) the number of other features with a correlation > 0.7 (favored the elimination of more features). In addition to the above criteria, one data set (protein-protein interactions) was eliminated because of a high frequency of missing values (88%). The synonymous substitution rate (*d*_*S*_) feature and any feature using *d*_*S*_ in their calculation were also excluded because they would be highly correlated with WGD timing and confound our analyses comparing the three WGD events. The filtering step left 11 features for building the general linear model. Following fitting the glm function, features were ranked according to their *p* values from the least to the greatest and the feature with the largest *p* value was dropped. The model was then fit to the reduced feature set and features were once again ranked. This process was repeated until the F-statistic (a measure of goodness of fit of the given model against a null model where all coefficients are set to zero) of the model was maximized and the final *p* value was calculated based on the maximal F-statistic. To evaluate the robustness of our models, we generated truncated versions of our data sets by leaving our one functional group and refitting the model, eliminating additional parameters if necessary to obtain the F-statistic maximizing models. Parameter estimates for the final model and each leave-one-out model can be found in Additional file 1: Tables S3-S5.

### Inferring ancestral expression levels and cis-regulatory sites

DNA-binding domains were identified in TF protein coding sequences using hmmscan via HMMER3 [44] based on the Pfam-A version 29.0 HMMs [18] with a threshold e-value of 1e-5. TFs were classified into families according to their DNA-binding domains and 44 of 59 TF families with ≥4 members were used for further analysis (Additional file 1: Table S11). For each TF family, full-length protein sequences were aligned using MAFFT [29] with default parameters. The phylogeny of each TF family was obtained using RAxML [66] with the following approach: rapid Bootstrapping algorithm, 100 runs, GAMMA rate heterogeneity, and the JTT amino-acid substitution model. These trees were then mid-point rooted with retree in PHYLIP [17]. Given the prevalence of duplication events and the tendency for TF duplicates to be retained in the plant lineages, homologs from other plants will be interlaced with TFs from *A. thaliana* in the phylogenies. This makes it challenging to hypothesize proper outgroup sequences. As such, we determined that midpoint rooting, while less than optimal, was the most consistent method we could apply across all TF family trees.

The mid-point rooted trees were used to infer the ancestral gene expression states and the *cis-*regulatory sites of WGD-duplicate TF pairs with BayesTrait [51] as was done in our earlier study [81]. Bayes Trait randomly assigns an evolutionary rate to the transition between possible states and uses these rates to determine mostly probably state of a given ancestral node. The likelihood of the observe states is then calculated and used to evaluate the current tree model and adjust evolutionary rates. This process is repeated iteratively to maximize the likelihood until either a maximum number of iterations or convergence is reached. This process is performed 100 times for each tree in order to evaluate the robustness of the inferred state and we only used ancestral states which were present in > 50 trees which is a non-trivial threshold as there are five possible states for each expression condition (each quantile and the ambiguous state). Further detail can be found at (http:// www.evolution.rdg.ac.uk/ BayesTraitsV2.0Files/TraitsV2Manual.pdf).

The expression data sets used are described in Additional file 1: Table S9. The discretized gene expression state (0,1,2,3) was based on the quartiles of gene expression levels within each experiment. Thus the inferred, ancestral expression state was also discretized. For *cis-*regulatory sites, the binding targets of 345 *A. thaliana* TFs were defined based DNA Affinity Purification-Sequencing data [48] from the Plant Cistrome Database (http://neomorph.salk.edu/dap_web/pages/index.php) where at least 5% of the read associated with a site were found to be in the 200 bp peak region. We inferred whether a site was present or absent (0,1) in the common ancestor of a duplicate pair. For both expression and regulatory site data, in cases where there was a missing value, it was explicitly included as an ambiguous state. To call the ancestral state from the expression or *cis-*regulatory site data, we required a posterior probability > 0.5. Cases where the called state was ambiguous or no majority existed were excluded from further analysis.

### Asymmetry of the retention of ancestral expression and regulatory sites

For determining expression state asymmetry, only TF WGD-duplicates with ≥5 partitioned ancestral expression states in one of the four expression datasets (Ctrl, LightDev, Stress, and Diff) were considered. For a WGD-duplicate pair with genes A and B, if the number of inherited ancestral expression states in A was larger or equal to that in B, then A and B were defined as the ancestral and the non-ancestral duplicate copies, respectively. The degree of asymmetry (*Y*_*A,B*_) of expression states between two duplicates was defined as:

$$ {Y}_{A,B}=\max \left({F}_A,{F}_B\right)-\left(1-\max \left({F}_A,{F}_B\right)\right) $$

Where *F*_*A*_ and *F*_*B*_ are the frequency with which ancestral expression was retained for duplicates A and B, respectively. By definition, *F*_*A*_ *+ F*_*B*_ = 1, such that *Y*_*A,B*_ has value between 0 (when F_A_ = F_B_, no asymmetry) and 1 (when either F_A_ or F_B_ = 1, maximum asymmetry).

With the asymmetry values for each TF pair, an average asymmetry value of all TF pairs was calculated for each expression dataset, as well as for the union of all TF duplicates from all datasets (1239 values total) to assess how the observed degree of asymmetry compared to what would be expected from if every partitioned state was independent (i.e. each gene has an equal chance of retaining the ancestral state regardless of the outcome of previous partitioning events). We also defined two subsets of the LightDev, Stress, and Diff data sets using the first and last element of each times series respectively because the expression of genes at different points of a time series are potentially correlated. The number of genes with > 5 partitioned conditions genes decreased in the subsets of LightDev (all = 334, first = 327, last = 325), Stress (all = 347, first = 265, last = 272), and Diff (all = 351, first = 277, last = 269) data sets. We excluded the Ctrl data set because it is composed of only four series, mean that no genes could pass the > 5 partitioned condition cutoff.

The expected distribution of asymmetry values for the expression states of TF WGD-duplicates (under the assumption of independent of partitioning events) was determined by conducting a series of Bernoulli trials equal to the total number of partitioned states amongst TF-WGD duplicates. In each of these trials there was an equal probability that either the first or second duplicate receive the ancestral state. The results of these trials were then grouped according the exact per gene distribution of partitioned states in TF-WGD duplicates and an asymmetry value was calculated for each group. This procedure was repeated 1000 times using an independent set of trials and subsequent groupings.

For assessing *cis-*regulatory site asymmetry, only TF WGD-duplicates with ≥5 inferred ancestral *cis-*regulatory sites we considered (402 WGD-duplicate pairs total). Similar to expression state asymmetry, in each duplicate pair the ancestral and non-ancestral duplicates were defined according to the number of inherited ancestral sites. For each WGD-duplicate pair, the degree of asymmetry of *cis-*regulatory site among a TF pair was defined analogous to what was done for expression. The expected distribution of asymmetry values for the *cis*-regulatory sites of TF WGD-duplicates was determined using the same procedure as for expression states.

### Ordinary differential equation models of TF state evolution

The change in expression states from the ancestral expression quartile to either a higher or lower quartile in an extant TF was modeled as a system of ordinary differential equations such that:

$$ \frac{d}{dt}\left(\begin{array}{c}O\\ {}+\\ {}-\end{array}\right)=\left(\begin{array}{ccc}-\left(x+y\right)& w& z\\ {}x& -w& 0\\ {}-y& 0& -z\end{array}\right)\left(\begin{array}{c}O\\ {}+\\ {}-\end{array}\right) $$

Where *O*, +, and - are the frequency of TF WGD duplicate genes retaining the ancestral expression states, having a higher-than-ancestral expression level, and having a lower-than-ancestral expression level, respectively. The parameters *x, y, w*, and *z* define the transition rates between these states. This system of equations was solved in Maxima (http://maxima.sourceforge.net/index.html) and best parameters for the observed distribution of duplicates pairs were determined using maximum likelihood estimates calculated with the bbmle package in R (https://cran.r-project.org/web/packages/bbmle/index.htmll). Non-linear minimization was used to approximate an initial guess, although the actual initial parameters often needed to be adjusted to reach a convergent solution. The best fit parameters for this single duplicate expression state evolution model can be found in Additional file 1: Table S12.

The loss of ancestral expression states in a pair of duplicated TFs was modeled as a system of ordinary differential equations such that:

$$ \frac{d}{dt}\left(\begin{array}{c}O\\ {}I\\ {} II\end{array}\right)=\left(\begin{array}{ccc}-x& y& 0\\ {}x& -\left(y+w\right)& z\\ {}0& w& -z\end{array}\right)\left(\begin{array}{c}O\\ {}I\\ {} II\end{array}\right) $$

Where *O, I,* and *II* are the frequency of TF WGD duplicate pairs where both, one, or neither duplicate retained the ancestral expression state. The parameters *x, y, w*, and *z* define the transition rates between these states. This system of equations was solved and the initial and best parameters were estimated in the same fashion as above. The best fit parameters for this pairwise expression state evolution model can be found in Additional file 1: Table S12. The same model was also applied to ancestral regulatory sites with *O, I,* and *II* representing the frequency of TF WGD duplicate pairs where both, one, or neither duplicate retained the ancestral regulatory site.

## Additional files

Additional file 1: **Table S1.** Lists of *Arabidopsis thaliana* genes in each GO category. **Table S2.** Variables considered for linear modeling and their sources. **Table S3.** Parameter Estimation for α WGD Retention Models. **Table S4.** Parameter Estimation for β WGD Retention Models**. Table S5.** Parameter Estimation for γ WGD Retention Models. **Table S6**. Number of experiments, samples, and inferred states from each expression data set. **Table S7.** Proportion of duplicate pairs with an expression state for each whole-genome duplication event and expression data set. **Table S8.** Average asymmetry of duplicate pairs across data sets. **Table S9.** List of experiments in each ATGenExpress dataset**. Table S10.** List of RNA-Seq Data Sets. **Table S11.** List of TFs in each Pfam domain family. **Table S12.** Initial conditions and inferred parameters for ODE models (XLSX 118 kb)

Additional file 2: **Figure S1.** Retention of WGD-duplicate genes in *A. thaliana.* The duplicate gene retention rates (log odds ratios) within 20 function groups relative to whole genome. Groups are ordered by the odds in the alpha event. Colors represent different WGD duplication events (α = orange, β = green, γ = blue). Bars indicated the 95% confidence interval of the odds of retention. If the confidence interval does not overlap with zero, this indicates the odds of retaining a duplicate gene is significantly different than the genome average from that function group at the 5% level. (PDF 46 kb)

Additional file 3: **Figure S2.** Difference between the observed rate of duplicate retention and rate predicted by the linear models of duplicate retention for each event (α = orange, β = green, γ = blue). Positive values indicate the observed rate is larger than the prediction while negatives values indicated the observed rate is less than the prediction. (PDF 289 kb)

Additional file 4: **File S1** Predicting WGD-duplicate retention status of individual genes using machine learning (DOCX 86 kb)

Additional file 5: **Figure S3** Difference in expression quartile of individual TF duplicates compared to their ancestral state for all four expression subsets (Control, LightDev, Diff, and Stress) across each WGD event (α = left, β = middle, γ = right). Heatmaps show the z-scores of the observed frequency of each difference compared to the expected frequency. Color correlates with the magnitude of the z-score, with darker red values indicated counts further above random expectation and dark blue values indicated counts further below random expectation. (PDF 536 kb)

Additional file 6: **Figure S4.** Deviation of pairs of TF WGD-duplicates from their ancestral state, defined as the difference value that each duplicated in a pair has from its ancestral state for all expression value subsets (Ctrl and Stress). Heatmaps show the z-scores scores of the observed frequency of each difference compared to the expected frequency. Color correlates with the magnitude of the z-score, with darker red values indicated counts further above random expectation and dark blue values indicated counts further below random expectation. (PDF 76 kb)

Additional file 7: **Figure S5.** ODE models of TF WGD-duplicate expression evolution relative to ancestral state for the Ctrl, Diff, and Stress expression subsets. In this mode, we consider the transition of the WGD-duplicate pair expression between three possible states relative to their ancestral state (O = both retained, I = one retained, II = neither retained). Results for one (left column) and two (right column) parameter models showing the change in time (x-axis) of the frequency (y-axis) of each WGD-duplicate-pair state (O = orange, I = blue, II = green). Curves represent the continuous output of the models while the symbols indicate the observed values on which the models were built (O = circle, I = square, II = triangle). (PDF 432 kb)

Additional file 8: **Figure S6.** ODE models of evolution of ancestral expression into either a higher or lower expression quartile from an ancestral expression state (O) to either a higher (+) or lower (−) expression state. Results for one (left column) and two (right column) parameter models show the change in time (x-axis) of the frequency (y-axis) of each state (O = orange, + = blue, − = green). Curves represent the continuous output of the models while symbols indicated the observed values on which the models were built (O = circle, + = square, − = triangle). (PDF 326 kb)

## Abbriviations

AUC-ROC: Area Under Curve-Receiver Operating Characteristic
TF: Transcription factor
WGD: Whole genome duplication
