# Supplementary material for: Expression and regulatory asymmetry of retained Arabidopsis thaliana transcription factor genes derived from whole genome duplication
Source: BMC Evol Biol. 2019 Mar 13;19:77. doi: 10.1186/s12862-019-1398-z (PMC6416927; doi:10.1186/s12862-019-1398-z)
Supplement: Supplementary file 2 — Figure S1. Retention of WGD-duplicate genes in A. thaliana. The duplicate gene retention rates (log odds ratios) within 20 function groups relative to whole genome. Groups are ordered by the odds in the alpha event. Colors represent different WGD duplication events (α = orange, β = green, γ = blue). Bars indicated the 95% confidence interval of the odds of retention. If the confidence interval does not overlap with zero, this indicates the odds of retaining a duplicate gene is significantly different than the genome average from that function group at the 5% level. (PDF 46 kb) [file 12862_2019_1398_MOESM2_ESM.pdf]

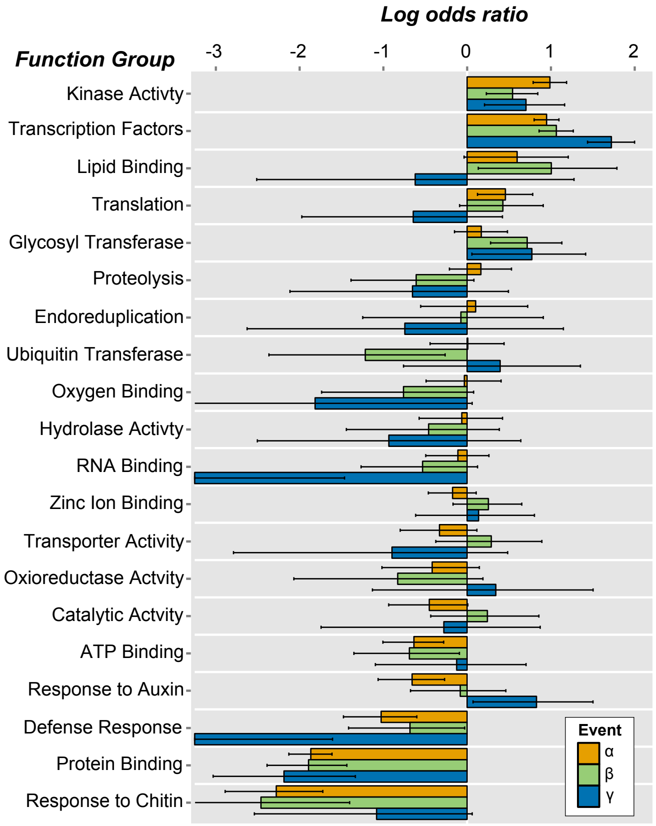

Figure S1 Retention of WGD-duplicate genes in A. thaliana. The duplicate gene retention rates (log odds ratios) within 20 function groups relative to whole genome. Groups are ordered by the odds in the alpha event. Colors represent different WGD duplication events (α = orange, β = green, γ = blue). Bars indicate the 95% confidence interval of the odds of retention. If the confidence interval does not overlap with zero, this indicates the odd of retaining a duplicate gene is significantly different than the genome average from that functional group at the 5% level.
