# Supplementary material for: Expression and regulatory asymmetry of retained Arabidopsis thaliana transcription factor genes derived from whole genome duplication
Source: BMC Evol Biol. 2019 Mar 13;19:77. doi: 10.1186/s12862-019-1398-z (PMC6416927; doi:10.1186/s12862-019-1398-z)
Supplement: Supplementary file 3 — Figure S2. Difference between the observed rate of duplicate retention and rate predicted by the linear models of duplicate retention for each event (α = orange, β = green, γ = blue). Positive values indicate the observed rate is larger than the prediction while negatives values indicated the observed rate is less than the prediction. (PDF 289 kb) [file 12862_2019_1398_MOESM3_ESM.pdf]

γ

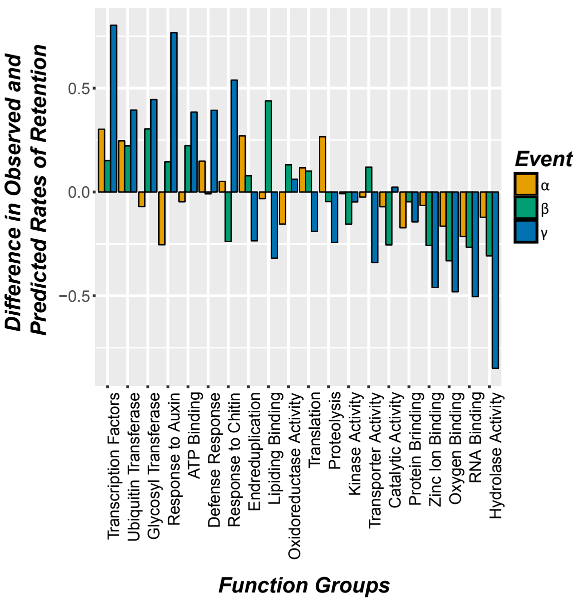

Figure S2. Difference between the observed rate of duplicate retention and rate predicted by the linear models of duplicate retention for each event (α = orange, β = green, γ = blue). Postive values indicate the observed rate is larger than the prediction while negative valeus indicate the observed rate is less than the prediction.
