# Supplementary material for: Expression and regulatory asymmetry of retained Arabidopsis thaliana transcription factor genes derived from whole genome duplication
Source: BMC Evol Biol. 2019 Mar 13;19:77. doi: 10.1186/s12862-019-1398-z (PMC6416927; doi:10.1186/s12862-019-1398-z)
Supplement: Supplementary file 4 — File S1 Predicting WGD-duplicate retention status of individual genes using machine learning (DOCX 86 kb) [file 12862_2019_1398_MOESM4_ESM.docx]

### Supplemental File S1. Predicting WGD-duplicate retention status of individual genes using machine learning

Machine learning models for TFs, kinases, and all genes in the genome were generated to predict whether a gene in a particular group had a retained WGD paralog from either the α, β, and γ event, as small numbers of β and γ made the difficult to correctly classify on their own. The machine learning was performed using the Random Forest algorithm implement in the R package randomForest (https://cran.r-project.org/web/packages/randomForest/index.html). We filtered the gene level feature set from a previous study [(Lloyd et al. 2015)](https://paperpile.com/c/WskiiM/Ms3r) by removing those with missing values for ≥5% of genes. For the remaining features, missing values were imputed with the rfImpute algorithm in randomForest using 10 iterations of 500 trees. The final matrix of genes and features for TFs, kinases, and the whole genome can be found in Tables S7, S8, and S9, respectively. Using the imputed data set for each group of genes and for each WGD event, we ran the Random Forest algorithm 10 times with 500 trees (each time with 10 fold cross validation) and collected the resulting votes (retained or not) for constructing Receiver Operating Characteristic curves (ROCs). The importance of each individual feature was assessed using Mean Decrease in Accuracy (MDA), the average number of genes misclassified across multiple runs as a result of removing the feature in question. The statistical significance of the difference in values of a feature between WGD-duplicates and WGD-singletons was determined using Welch’s t-test.

To evaluate the performance of our classifiers, we determined receiver operating characteristic curves (ROCs) for each model (Fig 1) and calculated the Area Under Curve (AUC-ROC), a metric that summarizes the ability of the classifier to recover true positive WGD-duplicate genes at different false positive rates. An AUC-ROC of 0.5 indicates that the classifier is no better than randomly labeling genes as having a retained duplicate or not, while an AUC-ROC of 1.0 indicates that the classifier can make predictions without error. Among the classifiers, the one characterizing the full genome performed best (AUC-ROC = 0.86), followed closely by protein kinases (AUC-ROC = 0.82), while the classifier for TFs, while much better than random, did not perform as well (AUC-ROC = 0.74). To investigate the source of the difference, we determined the importance of each feature to the classifier by calculating the Mean Decrease in Accuracy (MDA) which is the average number of genes misclassified across multiple runs as a result of removing a feature (Table 1). Given TFs are the least well predicted, we suspected the informative features for predicting retention in TFs would differ greatly from those for the genome at large and the protein kinases. Contrary to this expectation, the ranking of importance for TF WGD-duplicate prediction was more similar to the ranking of features for the whole genome prediction (Spearman’s rank, ρ = 0.86) than the ranking of features protein kinases to the whole genome prediction (Spearman’s rank, ρ = 0.51). This finding suggests that the feature value distributions of TF WGD-duplicate and WGD-singletons are more similar to the genome at large. Therefore, the reason that TF duplicate prediction model had lower performance was not simply because their feature values were substantially different from other duplicate genes. Instead, the features examined simply have lower importance in general for predicting TF retention (average MDA=11.3) than for other genes (average MDA=47.9), suggesting there are additional features important for TF retention that were not considered. For example, we might expect the number of DNA binding sites to be predictive of duplication status as an indication of the breadth of function of the TF which is related to the probability that a duplicate copy has been retained through subfunctionalization or gene balance.

Furthermore, the most informative feature for classifying kinases and the whole genome, the percent identity to the best matching paralog in A. thaliana, was less important when applied to TFs (Table 3). Although the maximum percent identity of WGD-duplicates compared to WGD-singletons is significantly higher in full genome (p = 1e-320), protein kinases (p = 1.1e-36), and TFs (p = 6.2e-12), the magnitude of the difference was greater for protein kinases (11.2%) and the whole genome (11.3%) than TFs (4.4%). This is due to WGD duplicate TFs having lower maximum percent identity (71.3%) than either kinases (75.2%, p=4.1e-24, t-test) or all genes (72.5%, p=5.9e-83 , t-test), while WGD-singletons TF had higher identity (66.9%) than kinases (64.0%, p=4.2E-35, t-test) and all genes (61.3%, p=1.9e-223, t-test). This observation may related to non-duplicate TF genes having apparent paralogs more often than non-duplicate genes do on average across the A. thaliana genome (Fig S2). The variance in the importance of maximum percent identity accounts for most of the performance difference across the classifiers as removing this feature yields similar results from all three (Fig S3). Similarly, inflating the difference in the percent identity of TF WGD-duplicates and WGD-singletons from 4.4% to 11.2% (the difference for protein kinases) would raise the predicted retention of TF from the γ WGD from 2.50 to 2.94, making up for more than half of the original error.

We would expect that other features used in our linear models would also be useful for classifying genes within function groups. However, the average importance rank of features found in more than one linear model was low (13.9 of 20), with the maximum expression value in RNA-seq being the worst feature in both the whole genome and TF classifiers. Of the four linear model features, mean expression in AtGenExpress had the highest rank in the whole genome (12th), TF (7th), and kinase classifiers (5th). However, the difference in mean expression between WGD-duplicates and WGD-singletons was not consistent: WGD duplicates genes were more highly expressed across the whole genome (+0.32, p=4.0e-23), and TFs (+0.37, p=1.0e-4), but in protein kinases WGD-singletons were more highly expressed, though not at a significant level (-1.1, p=0.77). Hence, not only does relationship between gene features and retention depend on the gene function, but the relationship within individual function groups can be the opposite direction of the relationship across function groups. For example, the high retention of the TF function group is in part due to relatively low average expression in AtGenExpress, but within TFs, genes with higher average expression are more often WGD duplicates. This suggests that selection for duplicate retention is dependent not only on function and features, but their interaction as well, though the exact nature of these interactions is beyond the scope of this study.

**Table 1**. The importance (rank) of all features used in the classification of individual duplicate genes

| **Feature** | **Genome^1^** | **Kinases^1^** | **TFs^1^** |
| --- | --- | --- | --- |
| Maximum Percent Identity (Paralog) | 171.6 (1) | 57.9 (1) | 29.4 (2) |
| Sequence Conservation (Viridiplantae) | 109.3 (2) | 27.2 (2) | 31.8 (1) |
| Gene Family Size (OrthoMCL) | 81.6 (3) | 2.0 (19) | 27.7 (3) |
| Protein Length (in Amino Acids) | 52.5 (4) | 14.2 (3) | 10.5 (11) |
| Expression Breadth (AtGenExpression) | 46.2 (5) | 9.4 (10) | 11.2 (8) |
| Expression MAD/Median (AtGenExpress) | 41.0 (6) | 5.4 (11) | 11.8 (5) |
| Expression Mean (LightDev Data) | 40.8 (7) | 10.4 (7) | 10.7 (9) |
| Expression Breadth (RNASeq) | 40.0 (8) | 3.3 (15) | 11.5 (6) |
| Expression Mean (Control Data) | 39.2 (9) | 10.7 (6) | 10.7 (10) |
| Expression Median (AtGenExpress) | 37.5 (10) | 10.2 (8) | 10.2 (12) |
| Expression Mean (Stress Data) | 37.0 (11) | 11.5 (4) | 12.0 (4) |
| Expression Mean (AtGenExpress) | 36.9 (12) | 11.3 (5) | 11.3 (7) |
| Expression Median (RNASeq) | 34.8 (13) | 4.7 (12) | 4.9 (16) |
| Sequence Conservation (Metazoa) | 34.5 (14) | 3.6 (13) | 4.4 (18) |
| Nucleotide Diversity (Pi) | 32.4 (15) | 9.7 (9) | 6.2 (14) |
| Expression Mean (Diff Data) | 31.6 (16) | 1.7 (2) | 7.9 (13) |
| Sequence Conservation (Fungi) | 30.6 (17) | 2.4 (17) | 4.6 (17) |
| Number of Protein Domains | 28.4 (18) | 2.3 (18) | 5.6 (15) |
| Expression Mean (RNASeq) | 18.6 (19) | 3.0 (16) | 2.9 (19) |
| Expression Maximum (RNASeq) | 12.9 (20) | 3.3 (14) | 0.4 (20) |

1: The importance of the feature as defined by the mean decrease in accuracy of the classification when the feature is removed. Features are ordered according to the rank of their importance in the whole genome model and the rank of each value for each model is indicated by ().

**References**

[Lloyd JP, Seddon AE, Moghe GD, Simenc MC, Shiu SH. 2015. Characteristics of Plant Essential Genes Allow for within- and between-Species Prediction of Lethal Mutant Phenotypes. *The Plant Cell* 27:2133–47.](http://paperpile.com/b/WskiiM/Ms3r)
