# Supplementary material for: Expression and regulatory asymmetry of retained Arabidopsis thaliana transcription factor genes derived from whole genome duplication
Source: BMC Evol Biol. 2019 Mar 13;19:77. doi: 10.1186/s12862-019-1398-z (PMC6416927; doi:10.1186/s12862-019-1398-z)
Supplement: Supplementary file 5 — Figure S3 Difference in expression quartile of individual TF duplicates compared to their ancestral state for all four expression subsets (Control, LightDev, Diff, and Stress) across each WGD event (α = left, β = middle, γ = right). Heatmaps show the z-scores of the observed frequency of each difference compared to the expected frequency. Color correlates with the magnitude of the z-score, with darker red values indicated counts further above random expectation and dark blue values indicated counts further below random expectation. (PDF 536 kb) [file 12862_2019_1398_MOESM5_ESM.pdf]

Ctrl

α

Z-Score	Extant Quartile			
Ancestral Quartile	1	2	3	4
1	73.09	-21.35	-33.56	-26.01
2	-18.53	59.145	-22.93	-21.95
3	-32.49	-14.91	62.192	-16.03
4	-28.28	-21.7	-6.233	68.632

β

Z-Score	Extant Quartile			
Ancestral Quartile	1	2	3	4
1	36.627	-8.711	-18.89	-14.79
2	-7.566	28.73	-11.85	-12.97
3	-17.51	-8.893	36.212	-7.971
4	-15.04	-11.03	-4.094	38.167

γ

Z-Score	Extant Quartile			
Ancestral Quartile	1	2	3	4
1	27.462	-7.049	-14.21	-13.25
2	-6.26	17.254	-4.057	-4.301
3	-12.87	0.9305	15.775	-2.281
4	-15.4	-2.937	3.6501	19.366

LightDev

α

Z-Score	Extant Quartile			
Ancestral Quartile	1	2	3	4
1	136.68	-38.78	-58.51	-45.62
2	-39.45	114.61	-44.83	-42.04
3	-54.77	-34.55	119.20	-30.39
4	-46.83	-42.62	-17.74	130.42

β

Z-Score	Extant Quartile			
Ancestral Quartile	1	2	3	4
1	72.43	-19.97	-32.89	-28.32
2	-17.90	60.76	-25.17	-27.24
3	-29.73	-15.65	65.66	-15.46
4	-28.99	-27.21	-4.47	76.19

γ

Z-Score	Extant Quartile			
Ancestral Quartile	1	2	3	4
1	49.99	-11.45	-23.35	-22.45
2	-14.16	27.10	-1.05	-11.15
3	-20.08	-0.28	32.82	-10.20
4	-23.34	-7.62	-6.77	43.39

Diff

α

Z-Score	Extant Quartile			
Ancestral Quartile	1	2	3	4
1	167.95	-34.254	-50.992	-91.975
2	-40.406	139.16	-19.555	-53.434
3	-49.056	-18.388	142.09	-52.85
4	-90.846	-58.062	-49.235	168.07

β

Z-Score	Extant Quartile			
Ancestral Quartile	1	2	3	4
1	80.056	-11.778	-20.633	-52.106
2	-16.474	63.81	-5.4744	-25.226
3	-19.987	-3.6608	64.109	-23.703
4	-51.867	-26.899	-20.578	82.372

γ

Z-Score	Extant Quartile			
Ancestral Quartile	1	2	3	4
1	39.74	-3.2307	-13.307	-26.843
2	-7.8741	26.272	-1.0811	-10.791
3	-16.066	1.0853	30.869	-8.1678
4	-23.428	-12.907	-8.2243	39.448

Stress

α

Z-Score	Extant Quartile			
Ancestral Quartile	1	2	3	4
1	189.66	-52.52	-80.10	-66.86
2	-51.09	157.18	-57.34	-59.30
3	-77.53	-44.51	158.36	-41.25
4	-68.98	-55.14	-23.59	173.74

β

Z-Score	Extant Quartile			
Ancestral Quartile	1	2	3	4
1	95.54	-24.92	-43.46	-41.62
2	-22.75	78.35	-29.81	-35.46
3	-39.59	-21.51	85.34	-16.48
4	-42.75	-32.74	-3.78	96.61

γ

Z-Score	Extant Quartile			
Ancestral Quartile	1	2	3	4
1	68.25	-11.42	-36.35	-34.62
2	-17.00	35.21	-1.39	-13.69
3	-28.66	-2.75	39.77	-5.52
4	-37.92	-8.49	5.50	51.75

Figure S3. Difference in expression quartile of individual TF duplicates compared to their ancestral state for all four expression subsets (Ctrl, LightDev, Diff, and Stress) across each WGD event (α = left, β = middle, γ = right). Heatmaps show the z-scores of the observed frequency of each difference compared to the expected frequency. Color correlates with the magnitude of the z-score, with darker red values indicating counts further above random expectation and darker blue values indicating counts further below random expectation.
