# Supplementary material for: Expression and regulatory asymmetry of retained Arabidopsis thaliana transcription factor genes derived from whole genome duplication
Source: BMC Evol Biol. 2019 Mar 13;19:77. doi: 10.1186/s12862-019-1398-z (PMC6416927; doi:10.1186/s12862-019-1398-z)
Supplement: Supplementary file 6 — Figure S4. Deviation of pairs of TF WGD-duplicates from their ancestral state, defined as the difference value that each duplicated in a pair has from its ancestral state for all expression value subsets (Ctrl and Stress). Heatmaps show the z-scores scores of the observed frequency of each difference compared to the expected frequency. Color correlates with the magnitude of the z-score, with darker red values indicated counts further above random expectation and dark blue values indicated counts further below random expectation. (PDF 76 kb) [file 12862_2019_1398_MOESM6_ESM.pdf]

Ctrl

α

Z-score (Duplicate 2 Deviat on)	Duplciate 1 Deviat on -3	-2	-1	0	1	2	3
-3	-0.51	-1.50	3.27	-0.15	n/a	n/a	n/a
-2		-1.83	-4.83	6.68	-2.59	n/a	n/a
-1			-8.42	13.33	-7.88	-2.54	n/a
0				-15.70	8.74	5.19	2.11
1					-1.68	-2.54	-1.05
2						-0.53	-0.41
3							-0.10

β

Z-score (Duplicate 2 Deviat on)	Duplciate 1 Deviat on -3	-2	-1	0	1	2	3
-3	-0.64	4.19	0.38	0.05	n/a	n/a	n/a
-2		0.17	-1.41	2.51	-3.99	n/a	n/a
-1			-0.88	5.15	-5.92	-1.36	n/a
0				-6.87	5.54	0.91	-0.15
1					0.74	-1.36	-0.93
2						-0.22	-0.22
3							-0.11

γ

Z-score (Duplicate 2 Deviat on)	Duplciate 1 Deviat on -3	-2	-1	0	1	2	3
-3	-0.51	-1.17	3.30	-0.64	n/a	n/a	n/a
-2		-1.27	3.57	0.30	-2.20	n/a	n/a
-1			-0.10	-1.43	-0.21	-1.46	n/a
0				0.25	2.43	-2.21	1.41
1					-0.60	-1.46	-0.52
2						-0.55	-0.27
3							0.00

Stress

α

Z-score (Duplicate 2 Deviat on)	Duplciate 1 Deviat on -3	-2	-1	0	1	2	3
-3	11.56	-2.42	7.79	-3.73	n/a	n/a	n/a
-2		-5.30	-12.23	18.58	-9.23	n/a	n/a
-1			-17.54	30.66	-20.61	-6.49	n/a
0				-39.83	28.04	12.17	3.17
1					-11.51	-6.49	-2.33
2						-1.56	-0.83
3							-0.21

β

Z-score (Duplicate 2 Deviat on)	Duplciate 1 Deviat on -3	-2	-1	0	1	2	3
-3.0	0.82	7.18	3.31	-0.98	n/a	n/a	n/a
-2.0		1.24	-1.15	4.33	-8.35	n/a	n/a
-1.0			-4.63	12.86	-15.43	-1.83	n/a
0.0				-15.31	13.03	-0.12	-2.37
1.0					2.03	-3.65	-1.62
2.0						-0.71	-0.41
3.0							-0.11

γ

Z-score (Duplicate 2 Deviat on)	Duplciate 1 Deviat on -3	-2	-1	0	1	2	3
-3.0	10.06	3.13	2.61	-4.13	n/a	n/a	n/a
-2.0		-1.74	1.01	4.24	-6.37	n/a	n/a
-1.0			6.42	-2.94	-4.48	-3.80	n/a
0.0				-0.81	2.59	2.19	-0.05
1.0					4.44	-3.80	-0.83
2.0						-0.83	-0.27
3.0							0.00

Figure S4. Deviation of pairs of TF WGD-duplicates from their ancestral state, defined as the difference value that each duplicate in a pair has from its ancestral state for all expression value subsets (Ctrl and Stress). Heatmaps show the z-scores of the observed frequency of the WGD-duplicate pair deviation compared to the expected frequency across all three duplicate events (α = top, β = middle, γ = bottom). Color correlates with the magnitude of the z-score, with darker red values indicating counts further above random expectation and darker blue values indicating counts further below random expectation.
