# Supplementary material for: Expression and regulatory asymmetry of retained Arabidopsis thaliana transcription factor genes derived from whole genome duplication
Source: BMC Evol Biol. 2019 Mar 13;19:77. doi: 10.1186/s12862-019-1398-z (PMC6416927; doi:10.1186/s12862-019-1398-z)
Supplement: Supplementary file 8 — Figure S6. ODE models of evolution of ancestral expression into either a higher or lower expression quartile from an ancestral expression state (O) to either a higher (+) or lower (−) expression state. Results for one (left column) and two (right column) parameter models show the change in time (x-axis) of the frequency (y-axis) of each state (O = orange, + = blue, − = green). Curves represent the continuous output of the models while symbols indicated the observed values on which the models were built (O = circle, + = square, − = triangle). (PDF 326 kb) [file 12862_2019_1398_MOESM8_ESM.pdf]

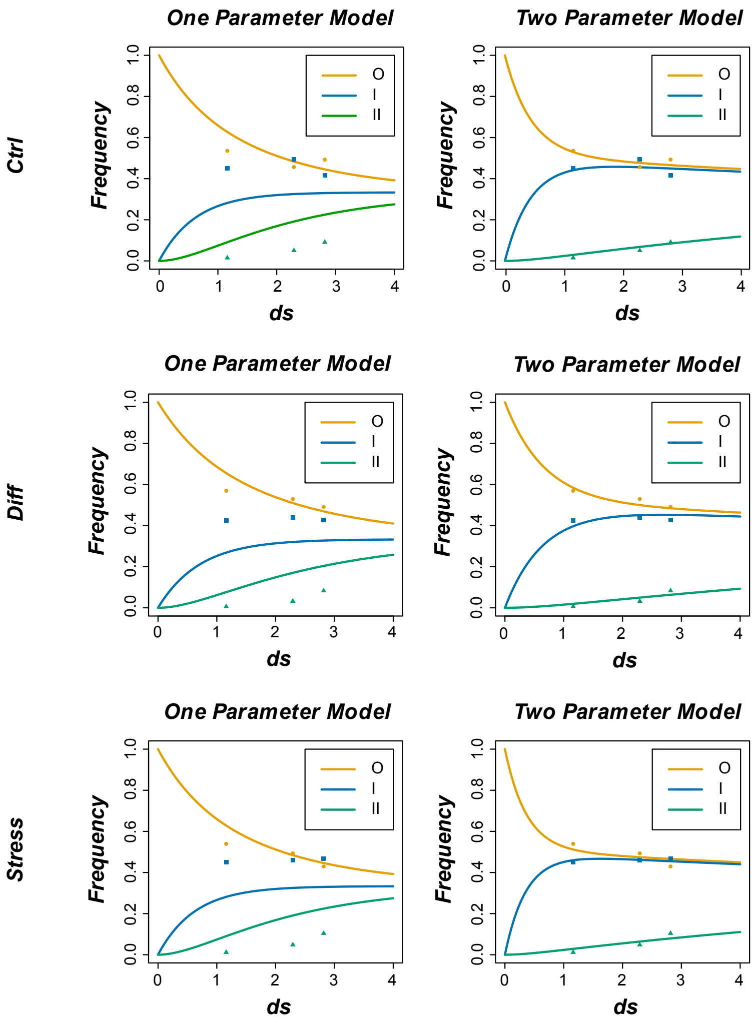

Figure S6. ODE models of TF WGD-duplicate expression evolution relative to ancestral state for the Ctrl, Diff, and Stress expression subsets. In this model, we consider the transition of the WGD-duplicate pair expression between three possible states relative to their ancestral state (O = both retained, I = one retained, II = neither retained). Results for one (left column) and two (right column) parameter models showing the change in time (x-axis) of the frequency (y-axis) of each WGD-duplicate-pair state (O = orange, I = blue, II = green). Curves represent the continuous output of the models while the symbols indicate the observed values on which the models were built (O = circle, I = square, II = triangle).
